# Two rare *PROX1* variants in patients with lymphedema

**DOI:** 10.1002/mgg3.1424

**Published:** 2020-08-05

**Authors:** Maurizio Ricci, Bruno Amato, Shila Barati, Rita Compagna, Dominika Veselenyiova, Sercan Kenanoglu, Liborio Stuppia, Tommaso Beccari, Mirko Baglivo, Danjela Kurti, Juraj Krajcovic, Roberta Serrani, Munis Dundar, Syed H. Basha, Pietro Chiurazzi, Matteo Bertelli

**Affiliations:** ^1^ Division of Rehabilitation Medicine Azienda Ospedaliero‐Universitaria Ospedali Riuniti di Ancona Italy; ^2^ Department of Clinical Medicine and Surgery University Federico II of Naples Naples Italy; ^3^ MAGI Euregio Bolzano Italy; ^4^ Department of Public Health University of Naples Federico II Naples Italy; ^5^ Department of Biology Faculty of Natural Sciences University of Ss. Cyril and Methodius In Trnava Trnava Slovakia; ^6^ Department of Medical Genetics Faculty of Medicine Erciyes University Kayseri Turkey; ^7^ Aging and Translational Medicine Research Center (CeSI‐MeT) University "G. d'Annunzio" Chieti‐Pescara Italy; ^8^ Department of Pharmaceutical Sciences University of Perugia Perugia Italy; ^9^ MAGI‐Balkan Tirana Albania; ^10^ Innovative Informatica Technologies Telangana India; ^11^ Istituto di Medicina Genomica Università Cattolica del Sacro Cuore Rome Italy; ^12^ Fondazione Policlinico Universitario “A.Gemelli” IRCCS UOC Genetica Medica Rome Italy; ^13^ EBTNA‐Lab Rovereto TN Italy; ^14^ MAGI's Lab Rovereto TN Italy

**Keywords:** genetic diagnosis, lymphedema, next‐generation sequencing (NGS), *PROX1*

## Abstract

**Background:**

The *PROX1* gene is specifically expressed in a subpopulation of endothelial cells that, by budding and sprouting, give rise to the lymphatic system. It also plays a critical role in neurogenesis and during development of many organs, such as the eye lens, liver, and pancreas.

**Methods:**

We used next‐generation sequencing (NGS) to sequence the DNA of a cohort of 246 Italian patients with lymphatic malformations. We first investigated 29 known disease‐causing genes: 235 of 246 patients tested negative and were then retested for a group of candidate genes, including *PROX1*, selected from a database of mouse models. The aim of the study was to define these patients’ genotypes and explore the role of the candidate gene *PROX1* in lymphedema.

**Results:**

Two of 235 probands were found to carry rare heterozygous missense variants in *PROX1*. In silico analysis of these variants—p.(Leu590His) and p.(Gly106Asp)—indicates that the overall protein structure was altered by changes in interactions between nearby residues, leading to functional protein defects.

**Conclusions:**

Our results suggest that *PROX1* is a new candidate gene for predisposition to lymphedema.

## Graphical abstract

Background: The *PROX1* gene is a specific marker expressed in a subpopulation of endothelial cells that by budding and sprouting give rise to the murine lymphatic system. It plays a critical role in embryo development and functions as a key regulatory protein in neurogenesis and the development of many organs, including the lymphatic system. Methods and Results: We used NGS to sequence the DNA of a cohort of 246 Italian patients with lymphatic malformations. We first investigated 29 known disease‐causing genes: 235 of 246 patients tested negative and were then tested for a group of candidate genes, including *PROX1*, selected from a database of mouse models. The aim of the study was to define these patients’ genotypes and explore the role of the candidate gene *PROX1* in lymphedema. Two of 235 probands were found to carry rare missense heterozygous variants in PROX1. In silico analysis suggested that the overall protein structure was somehow altered by changes in interactions between nearby residues, leading to functional protein defects. Conclusions: Our results suggest that PROX1 is a possible candidate for predisposition to lymphedema.

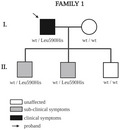

## 1 INTRODUCTION

Lymphedema is a disorder caused by malfunctioning of the lymphatic system with slow or blocked lymphatic flow and fluid build‐up in tissues (Michelini et al., 2017). Primary lymphedema has been associated with mutations in a number of genes (Michelini et al., 2018) and may be due to malformation of lymphatic vessels. Secondary lymphedema is caused by injury to the lymphatic system from surgical, physical, chemical, and infectious causes; however, several polymorphic DNA variants have been described that appear to predispose to secondary lymphedema (Michelini et al., 2018; Newman et al., 2012).

The prospero homeobox 1 gene *PROX1* (OMIM *601546) encodes a transcription factor that is conserved throughout vertebrates and plays an essential role during development, regulating cell fate determination and inducing progenitor cells in a number of organs (Oliver et al., 1993). PROX1 is found both in the cytoplasm and in the nucleus and functions as either a transcriptional activator or repressor, depending on the cellular and developmental environment. This 737‐amino acid long protein plays a critical role during embryogenesis and acts as a key regulator of neurogenesis as well as during the development of heart, eye lens, liver, pancreas, and lymphatic system (Wigle & Oliver, 1999). Wilting et al. (2002) developed antibodies against the PROX1 protein and investigated its expression in human tissues using immunofluorescence. These authors showed that PROX1 is a marker of lymphatic endothelial cells (LECs) in normal and pathological human tissues, co‐expressed with vascular endothelial growth factor receptor‐3 (VEGFR‐3, officially known as FLT4) and CD31 (Wilting et al., 2002). For example, in 19‐week‐old human fetuses, PROX1 and VEGFR‐3 were both expressed in lymphatic trunks and capillaries, with VEGFR‐3 located on the membrane of LECs, whereas PROX1 was found in the nucleus. PROX1 and VEGF‐C/VEGFR‐3 are therefore essential for the development (and maintenance) of human lymphatic system (Alitalo, Tammela, & Petrova, 2005).

In mice, *Prox1* is expressed specifically in a subpopulation of endothelial cells, which give rise to the lymphatic system by budding and sprouting. *Sox18* is also required for LEC differentiation, apparently because the Sox18 protein directly binds the *Prox1* promoter, inducing its transcription (François et al., 2008). In fact, *Sox18* is expressed first in a subset of vein cells that eventually co‐express *Prox1* and migrate to form lymphatic vessels (François et al., 2008). *Prox1*‐expressing endothelial cells were observed in the jugular vein of E10 mouse embryos and from there they migrate to form the first lymphatic sprouts. It has been shown that in *Prox1* null mice, budding and sprouting are altered and the structure and pattern of lymphatic vessels are disrupted, while angiogenesis and the vascular system are unaffected. Wigle and Oliver (1999) showed that *Prox1* ^+/−^ and *Prox1* ^−/−^ embryos begin to develop severe edema by midgestation (E14.5) and embryos homozygous for the disrupted *Prox1* allele die between E14.5 and E15.0. Heterozygous embryos (*Prox1* ^+/−^) at E14.5 showed a normal superficial lymphatic capillary network, which was completely absent in their homozygous (*Prox1* ^−/−^) littermates. These findings suggest that *Prox1* is a specific and necessary regulator of lymphatic system development and that the vascular and lymphatic systems develop independently (Wigle & Oliver, 1999). Further work by Johnson et al. (2008) showed that differentiation of LECs into blood endothelial cells (BECs) in mice is a plastic and reprogrammable process that depends on constant *Prox1* activity (Johnson et al., 2008). Indeed, small interfering RNA‐mediated downregulation of *Prox1* in cultured LECs or conditional downregulation of *Prox1* in the embryo is sufficient to reprogram LECs into BECs (Johnson et al., 2008). Actually, it has been found that *Prox1* levels are downregulated by *miR*‐*181a*, whose binding to the 3’UTR of *Prox1* mRNA results in rapid transcript degradation and reprogramming of LECs toward a blood vascular phenotype (Kazenwadel, Michael, & Harvey, 2010); as expected, *miR*‐*181a* levels are higher in embryonic BECs compared to LECs. Geng et al. (2016) further showed that formation of lymphovenous valves (LVVs) is severely disrupted in *Prox1* haploinsufficient embryos, contributing to the edema observed at murine midgestation. While downregulation of *Prox1* expression promotes loss of typical lymphatic cell junction characteristics (VE‐cadherin) and gain of blood vessel features (Johnson et al., 2008), *Prox1* overexpression induces differentiation of human adipose‐derived stem cells into lymphatic endothelial‐like cells (Deng et al., 2017).

*Prox1 *has also been associated with obesity related to lymphatic dysfunctions in mice (Harvey et al., 2005). In fact, these authors generated a *Prox1* conditional knock‐out mouse and found that functional inactivation of a single *Prox1* allele led to adult‐onset obesity due to abnormal lymph leakage from mispatterned and ruptured lymphatic vessels. The weight increase was due to subcutaneous and intra‐abdominal fat accumulation, most obviously in the fat pads around lymph nodes and in regions rich in lymphoid tissue like the mesentery (Harvey et al., 2005). After the initial suggestion that lymphatic dysfunction would lead to obesity in mice, findings in humans reinforced this hypothesis: genome‐wide association studies in Asian populations identified one single‐nucleotide polymorphism (SNP) (rs1704198) near the *PROX1* gene that is linked with increased waist circumference (Kim et al., 2013). Another SNP (rs340874) in the 5’UTR region of *PROX1 *has been associated with fasting hyperglycemia and type 2 diabetes (Adamska‐Patruno et al., 2019; Kretowski et al., 2015). Interestingly, subjects with the rs340874 CC‐genotype had higher accumulation of visceral fat despite lower daily food consumption (Kretowski et al., 2015). Finally, Surakka et al. (2015) showed that c.‐74G>A, a functional polymorphism in *PROX1*, is associated with increased triglyceride levels. All these results suggest that in a percentage of obese individuals, accumulation of abdominal fat may not be due to excessive calorie intake but could rather depend on a *PROX1* allelic variant that predisposes them to accumulation of abdominal fat. A further link between PROX1 and fatty acid metabolism has been found by Wong et al. (2017); in fact, PROX1 upregulates *CPT1A* expression, which encodes carnitine palmitoyltransferase 1A (CPT1A), a rate‐controlling enzyme in fatty acid β‐oxidation, and LEC‐specific loss of *Cpt1a*, in transgenic mouse models, decreases acetyl‐CoA production and impairs lymphatic development. Finally, Wong et al. (2017) also showed that PROX1 in human cells interacts with histone acetyltransferase p300 to increase transcription of lymphangiogenic genes.

While altered levels of PROX1 transcription factor have been reported in cancers of different organs (colon, brain, blood, breast, pancreas, liver, and esophagus), where they likely promote lymphangiogenesis, germinal variants of this gene have not yet been reported in association with human monogenic diseases.

Therefore, considering the importance of PROX1 in the development of lymphatic vessels, we decided to extend the analysis of our lymphedema patients who had turned out negative for a first set of genes, retesting them also for germinal variants of *PROX1*.

## 2 MATERIALS AND METHODS

### 2.1 Clinical evaluation

We analyzed 246 DNA samples from Caucasian patients diagnosed with primary lymphedema in hospitals across Italy. All patients were included retrospectively in our study and no consanguinity was reported in their families. The clinical diagnosis of lymphedema was confirmed by three‐phase lymphoscintigraphy according to the protocol of Bourgeois, Munck, Becker, Leduc, and Leduc (1997). Genetic testing was performed on germline DNA extracted from saliva or blood of each proband. When needed, segregation analysis was performed using DNA extracted from saliva of the available relatives.

### 2.2 Genetic analysis

A custom‐made oligonucleotide probe library was designed to capture all coding exons and flanking exon/intron boundaries (~15 bp) of 29 genes known to be associated with lymphedema. We then added the candidate gene *PROX1* to our panel.

Variants identified in proband DNA with clinical significance (pathogenic and likely pathogenic) and variants of unknown significance (VUS) according to the ACMG guidelines (Richards et al., 2015) were confirmed by bidirectional Sanger sequencing on a CEQ8800 Sequencer (Beckman Coulter). We developed a next‐generation sequencing (NGS) protocol for screening the most frequently mutated genes, namely *ADAMTS3* (OMIM *605011), *CELSR1* (OMIM *604523), *EPHB4* (OMIM *600011), *FAT4* (OMIM *612411), *FLT4* (OMIM *136352), *FOXC2* (OMIM *602402), *GATA2* (OMIM *137295), *GJA1* (OMIM *121014), *GJC2* (OMIM *608803), *HGF* (OMIM *142409), *KIF11* (OMIM *148760), *PIEZO1* (OMIM *611184), *PTPN14* (OMIM *603155), *SOX18* (OMIM *601618), and *VEGFC* (OMIM *601528), including the candidate gene *PROX1* (OMIM *601546). When each variant was found, we searched for it in the dbSNP database (www.ncbi.nlm.nih.gov/SNP/) and in the Human Gene Mutation Database professional (HGMD; http://www.biobase‐international.com/product/hgmd). In silico evaluation of the pathogenicity of all exonic variants was performed using the Variant Effect Predictor tool (http://www.ensembl.org/Tools/VEP) and MutationTaster (http://www.mutationtaster.org). Finally, minor allele frequencies (MAFs) were checked in the Genome Aggregation Database (GnomAD) (http://GnomAD.broadinstitute.org/) and all variants were evaluated according to American College of Medical Genetics and Genomics guidelines (Richards et al., 2015). Detailed pretest genetic counseling was provided to all subjects, who were then invited to sign a dedicated informed consent form in order to use their anonymized genetic results for research.

### 2.3 PROX1 protein structure prediction

The primary amino acid sequence of PROX1 in FASTA format (NP_001257545.1) was used to search for template libraries in the Swiss model template library (SMTL) version 2019‐10‐24 and Protein Data Bank (PDB) release 2019‐10‐18 (Berman et al., 2000) with BLAST (Camacho et al., 2009) and HHBlits (Remmert, Biegert, Hauser, & Söding, 2012) for evolution‐related structures matching the given PROX1 sequence. Models based on the target‐template alignment were built using ProMod3 of the SWISS‐MODEL server (Waterhouse et al., 2018). Coordinates conserved between the target and the template were copied from the template to the model, insertions and deletions were remodeled using a fragment library, and side chains were then rebuilt. Finally, the geometry of the resulting model was regularized using CHARMM27 force field (Mackerell, Feig, & Brooks, 2004). If loop modeling with ProMod3 failed, an alternative model was built with PROMOD‐II (Guex, Peitsch, & Schwede, 2009). Global and per‐residue model quality was assessed using the QMEAN scoring function (Benkert, Biasini, & Schwede, 2011). BioVia Discovery Studio Visualizer v17.2 was used to visualize the modeled protein, to mutate the targeted amino acids, and to analyze the molecular level interactions (Dassault Systèmes BIOVIA, 2017).

## 3 RESULTS

### 3.1 Clinical and genetic assessment

**Figure 1:**
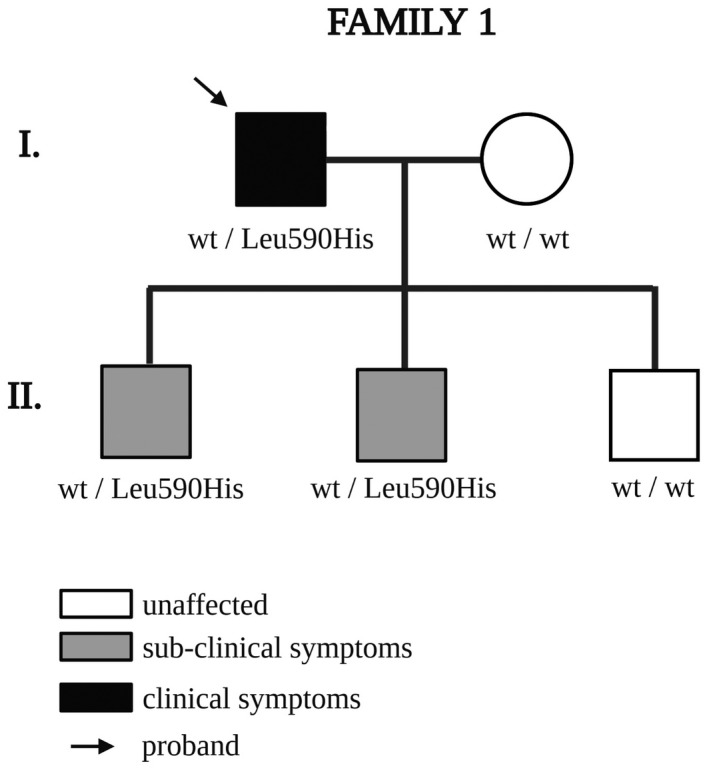
Pedigree of Family 1

In two of the 235 patients, who tested negative at the first genetic test, we identified two heterozygous missense variants in *PROX1*. Both cases were sporadic and apparently had no family history of lymphedema. Their clinical features are summarized in Table 1. The first proband was a 70‐year‐old man with late‐onset upper limb lymphedema at the age of 65 years; his three sons are reportedly not affected but two inherited the *PROX1* variant (Figure 1). Although these two sons (II‐1 and II‐2) have no clinical signs of lymphedema, both have subclinical symptoms of intermittent edema. The nomenclature of the variant is NM_001270616.1:c.1769T>A on cDNA, resulting in the missense change NP_001257545.1:p.(Leu590His) in the protein, where a highly conserved hydrophobic leucine is substituted by a hydrophilic histidine. Prediction programs such as MutationTaster, SIFT and PolyPhen, classify it as "disease‐causing," “deleterious,” and “probably damaging”, respectively (Table 2). This change falls just within the C‐terminal homeobox domain (spanning amino acids 579‐730), and it is not listed in dbSNP nor found in the GnomAD database collecting variants of the general population.

**Table 1:** Clinical features and *PROX1* genotype of presently reported patients

Family	Pedigree	Sex	Age	Clinical features	Age of onset	Familiarity	Genotype
1	Proband	M	70 years	Upper limbs lymphedema	65 years	NO	p.(Leu590His)/wt
1	Son 1	M	37 years	healthy	/	son	p.(Leu590His)/wt
1	Son 2	M	39 years	healthy	/	son	p.(Leu590His)/wt
1	Son 3	M	32 years	healthy	/	son	wt/wt
2	Proband	F	66 years	Left ankle and foot edema (after insect bite)	30 years	NO	p.(Gly106Asp)/wt

**Table 2:** Features of the two missense *PROX1* variants

Variants	dbSNP	MutationTaster	Affected domains	SIFT	PolyPhen	Frequency
*PROX1*:NM_001270616.1: c.1769T>A: NP_001257545.1: p.(Leu590His)	Not listed	Prediction: disease‐causing; highly conserved residue	DNA_BIND Prospero‐type homeobox; HELIX	deleterious	probably damaging	/
*PROX1*:NM_001270616.1: c.317G>A: NP_001257545.1: p.(Gly106Asp)	rs773237135	Prediction: disease‐causing; highly conserved residue	/	tolerated	probably damaging	0.0000159 (GnomAD)

The other proband is a 66‐year‐old woman with sporadic left ankle and foot edema. No other family member was available for testing. The onset of lymphedema occurred at age 30 years after an insect bite. The cDNA nomenclature of her variant is NM_001270616.1:c.317G>A resulting in the missense protein change NP_001257545.1:p.(Gly106Asp) that involves a very conserved hydrophobic glycine, that is changed into a hydrophilic aspartic acid. Prediction programs such as MutationTaster, SIFT, and PolyPhen classify it as "disease‐causing," “tolerated,” and “probably damaging”, respectively. This variant is listed in dbSNP (rs773237135) but its frequency in GnomAD is very low (Table 2), having been found in just four individuals (all females) of 125637.

### 3.2 Template selection and model building

**Figure 2:**
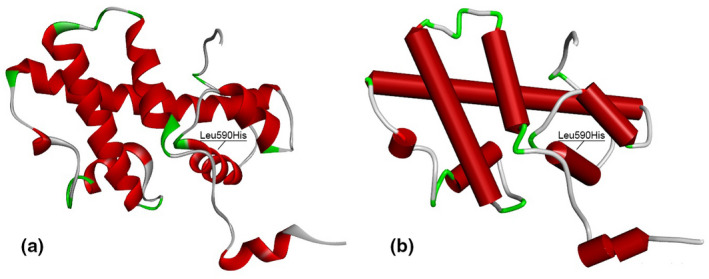
Modeled structure of PROX1 protein represented in (a) ribbon and (b) schematic. The helix domain affected by the Leu590His variant is indicated. White regions represent loops and red regions represent alpha helices

**Figure 3:**
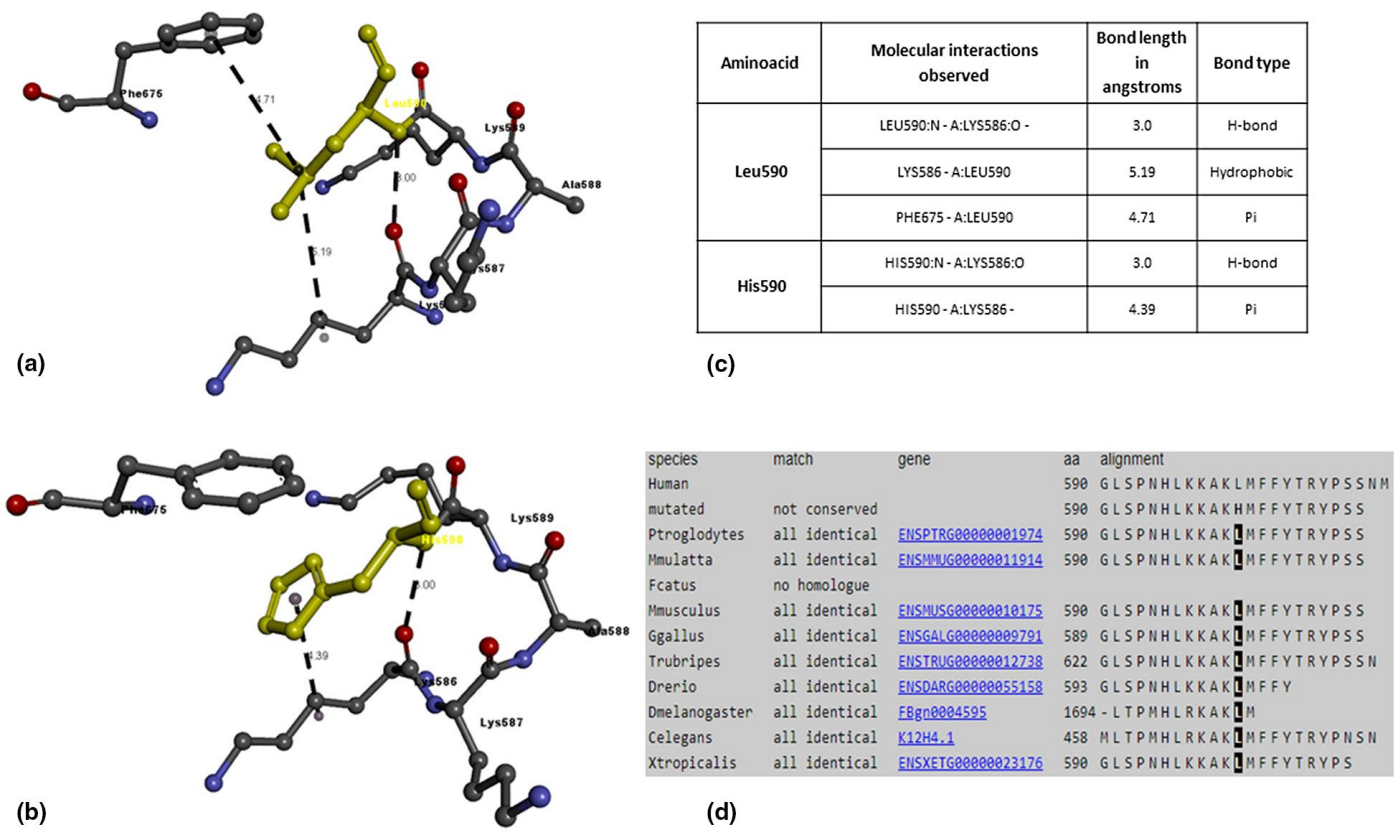
Molecular interactions of (a) Leu590 and (b) His590 (highlighted in yellow) of the modeled PROX1 protein with adjacent interacting residues and details of (c) molecular interactions (bond features) and (d) conservation of the amino acid residue

**Figure 4:**
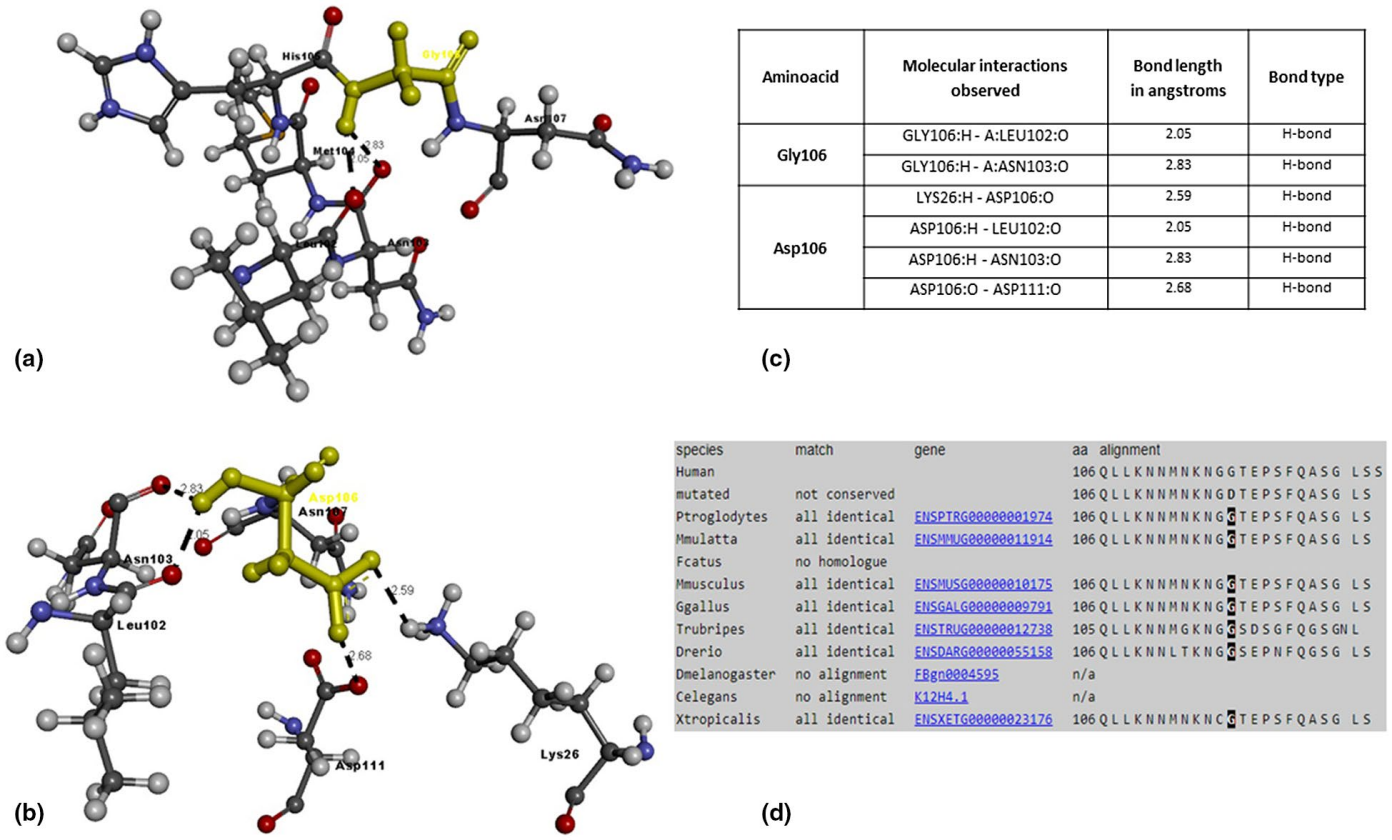
Molecular interactions of (a) Gly106 and (b) Asp106 (highlighted in yellow) of the modeled PROX1 protein with adjacent interacting residues and details of (c) molecular interactions (bond features) and (d) conservation of the amino acid residue

The PROX1 amino acid sequence (NP_001257545.1) was submitted to the SWISS‐MODEL server using BLAST and produced a total of 31 matching templates with variable sequence identity and quality percentages. Table 3 lists the details of the top 10 templates. A further 21 templates, which were considered less suitable for modeling, were also found: 6n6s.1.A, 6n6s.1.B, 6n6s.1.C, 6n6s.1.D, 5d60.1.A, 5d60.1.B, 5d60.1.C, 5d60.1.D, 6g6h.1.B, 6g6h.1.D, 6g6h.1.C, 4uot.1.A, 5d5z.2.A, 5d5z.2.B, 6g6h.1.A, 5d5y.1.A, 5d5y.1.B, 6g6h.1.E, 5d5z.1.C, 5d5z.1.B, and 5d5z.1.A. Based on the percentage of sequence identity, similarity, and best quality square, 2LMD chain was selected to align the template and query sequences for model building purposes. The resulting model is shown in Figure 2. The Discovery Studio visualizer was used to generate the molecular version of the mutated PROX1 protein with either missense variant (Gly106Asp and Leu590His). Molecular level interaction analysis was then performed to compare the wild‐type and mutated residues and their interactions. Snapshots of the wild‐type and mutant proteins are shown in Figures 3 and 4, along with details of the interacting residues (including type of bonds and bond lengths in Ångströms). The degree of residue conservation can also be appreciated by looking at panel d) of these two last figures; glycine 106 is conserved in all examined vertebrate species (Figure 4d) and leucine 590 is conserved also in *Drosophila* and *C*.* elegans* (Figure 3d).

**Table 3:** Best suited models for 3D modeling of PROX1 protein

Template	Sequence Identity	Oligo‐state	QSQE	Found by	Method	Resol.	Sequence similarity	Coverage	Description
2lmd.1.A	100.00	monomer	—	BLAST	NMR	NA	0.61	0.22	Prospero homeobox protein 1
2lmd.1.A	100.00	monomer	—	BLAST	NMR	NA	0.61	0.22	Prospero homeobox protein 1
1xpx.1.C	62.58	monomer	—	BLAST	X‐ray	2.80 Å	0.49	0.21	Protein prospero
1mij.1.A	63.09	monomer	—	BLAST	X‐ray	2.05 Å	0.50	0.20	Protein prospero
1xpx.1.C	61.01	monomer	—	HHblits	X‐ray	2.80 Å	0.49	0.22	Protein prospero
1mij.1.A	61.84	monomer	—	HHblits	X‐ray	2.05 Å	0.49	0.21	Protein prospero
6n6r.1.B	32.00	homo‐dimer	0.03	HHblits	X‐ray	1.95 Å	0.38	0.03	TNFAIP3‐interacting protein 1
6n6r.1.C	32.00	homo‐dimer	0.03	HHblits	X‐ray	1.95 Å	0.38	0.03	TNFAIP3‐interacting protein 1
6n5m.1.B	33.33	homo‐dimer	0.02	HHblits	X‐ray	3.01 Å	0.38	0.03	TNFAIP3‐interacting protein 1
6n5m.1.C	33.33	homo‐dimer	0.02	HHblits	X‐ray	3.01 Å	0.38	0.03	TNFAIP3‐interacting protein 1

## 4 DISCUSSION

Lymphedema occurs when the lymphatic system, consisting of thin vessels and lymph nodes, is damaged, blocked, or malformed. Lymphedema may be secondary to trauma (fractures and wounds) and cancer treatments (surgery and radiation therapy), but also inflammation (rheumatic diseases and phlebitis) and infections (recurrent cellulitis and filariasis) may damage and block lymph nodes and lymphatic vessels (Li, Kataru, & Mehrara, 2020). It has been suggested that secondary lymphedema cannot be attributed exclusively to environmental insults and that genetic susceptibility is a real possibility (Finegold et al., 2012).

Primary lymphedema is due to a malformation of the lymphatic system and has a genetic basis. Although the malformation is inborn, lymphedema is not always present at birth but it usually manifests in early adulthood (before the age of 30); however, presentation later in life is not uncommon (Azhar, Lim, Tan, & Angeli, 2020). Women are affected more often than men and this condition is usually more pronounced on one side and to the distal part of limbs. Primary lymphedema is often characterized by autosomal dominant transmission with several responsible loci, reduced penetrance, and variable expressivity. According to the Online Mendelian Inheritance in Man database (www.omim.org), variants in six genes have been associated with this phenotype (*CALCRL*, *EPHB4*, *GJC2*, *FLT4*, *PIEZO1*, and *VEGFC*); other genes responsible for inherited syndromes that include lymphedema among their clinical signs are also listed in OMIM. In 2016, we first reported a genetic screening in a cohort of Italian patients (45 familial and 71 sporadic) affected by primary lymphedema (Michelini et al., 2016) using an NGS panel with 10 genes associated with lymphatic diseases. In 2018, we reviewed all available genetic tests for lymphatic vascular malformations and lymphedema (Michelini et al., 2018) and extended our NGS panel including a further group of potential candidate genes, selected from the database of mouse models. With this improved approach, we sequenced a new cohort of 246 Italian patients with lymphatic malformations and identified 11 variants (data not shown). We then re‐evaluated the remaining 235 patients who were negative after the first‐tier analysis and run a second test in order to ascertain the potential role of other candidate genes, including *PROX1*.

In fact, we know from murine models that *Prox1* is expressed in a subpopulation of endothelial cells, which give rise to the lymphatic system by budding and sprouting. In *Prox1* null mice, vasculogenesis and angiogenesis are unaffected, but budding and sprouting of lymphatic vessels are blocked (Wigle & Oliver, 1999). The formation of lymphatic valves is also impaired in the *Prox1* haploinsufficient murine models, suggesting a role for this gene in lymphovenous drainage (Geng et al., 2016). In fact, downregulation of *Prox1* expression results in loss of typical lymphatic cell junction characteristics (VE‐cadherin) and gain of blood vessel features (pericytes and platelets) (Johnson et al., 2008). On the other hand, overexpression of *PROX1* in human adipose‐derived stem cells induces differentiation toward lymphatic endothelial lineage (Deng et al., 2017). Given the role in murine models of *Prox1* in the accumulation of adipose tissue associated with abnormal lymph leakage (Harvey et al., 2005), we thought it would be interesting to consider this gene in lymphedema screening.

Lymphedema is often associated or confused with the accumulation of fatty tissue and obesity. This is interesting if one considers the role of lymphatic vasculature in regulating energy metabolism (Ho & Srinivasan, 2020). Escobedo and Oliver (2017) also suggested that some SNPs located near or within genes associated with lymphatic development may predispose to adult‐onset obesity in humans. For example, rs1704198 near the *PROX1* gene seems associated with increased waist circumference (Kim et al., 2013) and rs340874 in the 5’UTR of *PROX1* predisposes to abdominal fat and is associated with fasting hyperglycemia and type 2 diabetes (Adamska‐Patruno et al., 2019; Kretowski et al., 2015). However, to our knowledge, no *PROX1* variants have yet been described in association with lymphedema.

Our results show that two of 235 probands, who were negative with our first‐tier genetic test, carried rare heterozygous missense variants in *PROX1*. Both cases appeared sporadic, although in the first family (Figure 1), the proband and two of his three sons carry the same missense *PROX1* variant. These sons are reportedly healthy, but they show lymphatic system abnormalities and intermittent edema. This missense variant (p.Leu590His) changes a very conserved protein residue, altering its nature (from hydrophobic to hydrophilic) and the three‐dimensional structure of the C‐terminal homeobox domain (see Figure 3). In silico analysis showed that wild‐type PROX1 is more stable with Leu590 forming three types of interaction with Lys586 and Phe675 residues (a hydrogen bond, a hydrophobic interaction, and a Pi‐interaction), while the His590‐mutated PROX1 is less stable due to loss of a crucial interaction with Phe675. Furthermore, prediction tools classify this variant as “disease‐causing,” “deleterious,” and “probably damaging”, respectively (Table 2). Finally, the p.Leu590His variant is not found in dbSNP and has no listed frequency in normal populations (GnomAD); however, segregation analysis confirmed that two of the three sons (who show occasional signs of edema) inherited the *PROX1* variant.

Our second proband is a 66‐year‐old woman with sporadic edema of the left foot and ankle, which reportedly begun after an insect bite when she was 30. The c.317G>A variant results in a missense change involving a conserved hydrophobic residue (glycine 106) that is converted into a hydrophilic one (aspartic acid). In silico analysis showed that the wild‐type Gly106 forms two hydrogen bonds with Leu102 and Asn103 residues, while the Asp106 mutant residues are predicted to have two more interactions with Lys26 and Asp111, causing an overall conformation change of PROX1 structure (see Figure 4). Finally, although dbSNP lists this variant (rs773237135), its frequency in the GnomAD database is extremely low (0.0000159) and compatible with a low penetrance allele.

Although both variants are presently classified as VUS according to the American College of Medical Genetics and Genomics and the Association for Molecular Pathology guidelines (Richards et al., 2015; https://varsome.com/), considering their extreme rarity in the general population and the concordant results of bioinformatics prediction tools as well as our own molecular modeling of mutant PROX1 protein, we propose that these two missense variants, identified in our patients, result in at least partial loss‐of‐function of PROX1 and predispose to lymphedema. In conclusion, the results of our study are in line with the hypothesis that heterozygous variants of *PROX1* caused predisposition to lymphedema in our patients and that *PROX1* should be considered as a new candidate gene for lymphedema in humans.

## CONFLICT OF INTEREST

No competing financial interests exist.

## AUTHOR CONTRIBUTIONS

MBe designed the work. SHB provided bioinformatics data. SM and BA provided the clinical information of the patient. SM, BA, SB, RC, and RS compiled the summary of clinical information of the cases. DV and SK performed detailed literature review and developed first draft. All authors offered a major contribution in the writing of the manuscript. LS, TB, JK, and MD provided critical feedback on the manuscript. All authors approved the final version.
